# The Role of Asymmetric Dimethylarginine (ADMA) in Endothelial Dysfunction and Cardiovascular Disease

**DOI:** 10.2174/157340310791162659

**Published:** 2010-05

**Authors:** Latika Sibal, Sharad C Agarwal, Philip D Home, Rainer H Boger

**Affiliations:** 1 Institute of Cellular Medicine, Newcastle University, Newcastle upon Tyne, UK; 2 Institute of Experimental and Clinical Pharmacology and Toxicology, University Medical Center Hamburg-Eppendorf, Hamburg, Germany

**Keywords:** Asymmetric dimethylarginine, endothelial, cardiovascular disease.

## Abstract

Endothelium plays a crucial role in the maintenance of vascular tone and structure. Endothelial dysfunction is known to precede overt coronary artery disease. A number of cardiovascular risk factors, as well as metabolic diseases and systemic or local inflammation cause endothelial dysfunction. Nitric oxide (NO) is one of the major endothelium derived vaso-active substances whose role is of prime importance in maintaining endothelial homeostasis. Low levels of NO are associated with impaired endothelial function. Asymmetric dimethylarginine (ADMA), an analogue of L-arginine, is a naturally occurring product of metabolism found in human circulation. Elevated levels of ADMA inhibit NO synthesis and therefore impair endothelial function and thus promote atherosclerosis. ADMA levels are increased in people with hypercholesterolemia, atherosclerosis, hypertension, chronic heart failure, diabetes mellitus and chronic renal failure. A number of studies have reported ADMA as a novel risk marker of cardiovascular disease. Increased levels of ADMA have been shown to be the strongest risk predictor, beyond traditional risk factors, of cardiovascular events and all-cause and cardiovascular mortality in people with coronary artery disease. Interventions such as treatment with L-arginine have been shown to improve endothelium-mediated vasodilatation in people with high ADMA levels. However the clinical utility of modifying circulating ADMA levels remains uncertain.

## INTRODUCTION

Atherosclerotic disease is a major cause of mortality around the world. Conventional cardiovascular risk factors like hypercholesterolemia, diabetes, smoking and hypertension can account for 80% of increased risk of coronary artery disease [1, 2].

The endothelium plays a crucial role in the maintenance of vascular tone [3]. Endothelium-derived mediators play a crucial role in vascular homeostasis. Endothelium-derived nitric oxide (NO) is a potent endogenous vasodilator. NO is formed in the endothelium by the endothelial isoform of nitric oxide synthase (NOS) [4]. It is released in response to shear stress and plays an important role in flow-mediated dilatation [5]. Besides inducing vasodilatation, NO inhibits aggregation of platelets [6], inhibits adhesion of monocytes and leukocytes to the endothelium [7], inhibits smooth muscle cell proliferation [8] and inhibits oxidation of LDL [9]. Endothelium-derived NO also inhibits vascular inflammation by suppressing the expression and activity of adhesion molecules and chemokines [10].

Together, these functions make NO a significant endogenous anti-atherosclerotic molecule. A reduction in NO can result in endothelial dysfunction and in an increased risk for cardiovascular disease. Endothelial dysfunction precedes overt atherosclerotic disease. Previous studies have shown endothelial dysfunction to predict the presence of cardiovascular disease and future cardiovascular events [11, 12].

In 1992, Vallance and co-workers reported that asymmetric dimethylarginine (ADMA) is a naturally occurring endogenous inhibitor of nitric oxide (NO) synthase [13]. ADMA reduces NO production and consequently could thus lead to endothelial dysfunction and cardiovascular events. An increased understanding of the pathophysiology of atherosclerosis, particularly of the central role of endothelial dysfunction, has led to the emergence of plasma ADMA as a putative cardiovascular risk marker.

Studies have shown that the increased concentrations of ADMA found in some pathophysiological conditions are associated with other factors giving increased risk of atherosclerosis such as increasing age, hypercholesterolemia, hypertension, hypertriglyceridemia, diabetes mellitus, insulin insensitivity, hyperhomocysteinemia and renal failure [14-21]. Furthermore, elevated plasma ADMA concentration has been identified as an independent risk factor for progression of atherosclerosis, cardiovascular death and all-cause mortality [22-24].

## ADMA BIOSYNTHESIS, METABOLISM AND EXCRETION

### Biosynthesis

**Fig. (1):**
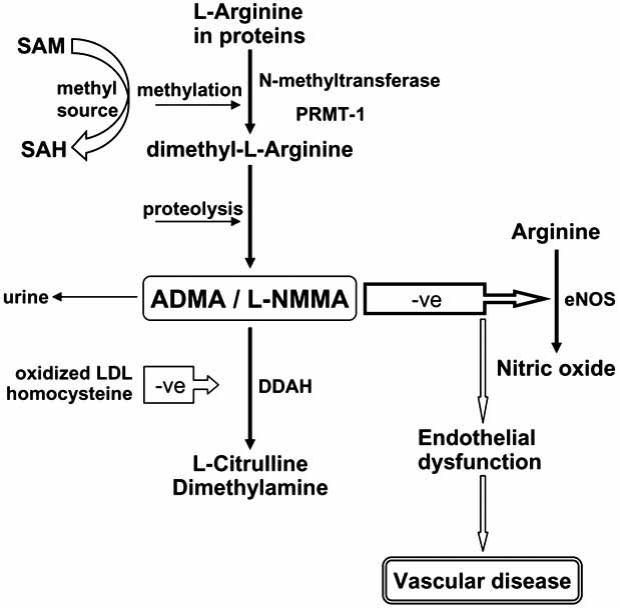
Overview of pathways of synthesis and metabolism of ADMA. Methylation of arginine residues within peptides occurs through N-methyltransferases, protein arginine N-methyltransferase-1 (PRMT-1). S-adenosylmethionine (SAM) is the methyl donor, changing to S-adenosylhomocysteine (SAH). Proteolytic breakdown of the proteins leads to the generation of ADMA and N-monomethyl-L-arginine (L-NMMA) within cells, and is detectable in the circulation. ADMA is an inhibitor of endothelial nitric oxide synthase (eNOS) by competing with its substrate L-arginine thus impairing nitric oxide (NO) production, thus leading to endothelial dysfunction and subsequently atherosclerosis. ADMA is eliminated partly via urinary excretion but mainly via metabolism by the enzyme dimethylarginine dimethylaminohydrolase (DDAH) to citrulline and dimethylamine.

Dimethylarginines are the result of the degradation of methylated proteins [25]. The methyl groups are derived from S-adenosylmethionine, with involvement of the enzymes protein arginine methyltransferase type 1 and 2 (PRMT1, PRMT2). PRMT-1 catalyses the formation of NG-monomethyl-L-arginine (LNMMA) and NG,NG-dimethyl-L-arginine (ADMA), while PRMT-2 methylates proteins to release NG,N'G-dimethyl-L-arginine (symmetric dimethyl- arginine; SDMA) and L-NMMA [26, 27]. The asymmetri-cally methylated arginine residues (L-NMMA and ADMA), but not symmetrically methylated arginine (SDMA), are compe-titive inhibitors of the nitric oxide synthases. The release of ADMA from endothelial cells is increased in the presence of native or oxidized LDL, possibly mediated by up-regulation of S-adenosylmethionine dependent methyl transferases [28] (Fig. **1**). Moreover, recent data have shown that the lung appears to contain large amounts of protein-bound ADMA, due to the high expression levels of various PRMTs in lung tissue [29]. 

### Metabolism and Excretion

Renal excretion plays a role in the elimination of endogenous ADMA and SDMA. Urinary excretion of SDMA in rabbits has been shown to be 30 times greater than that of either L-NMMA or ADMA [25]. A number of studies have reported increased levels of ADMA and SDMA in people with renal failure [21, 30]. Interestingly, haemodialysis leads to a lower clearance of ADMA than predicted, suggesting there are alternative non-renal route(s) of removal of circulating ADMA [31].

The specific pathway involved in metabolism of ADMA but not SDMA occurs via hydrolytic degradation to citrulline and dimethylamine catalysed by the enzyme called NG dimethylarginine dimethylaminohydrolase (DDAH) [32] (Fig. **1**).

DDAH activity has been found in kidney, pancreas, liver, brain and aorta with immunoexpression also in neutrophils and macrophages [33, 34]. Inhibition of DDAH causes gradual vasoconstriction which is reversed by L-arginine [35]. There are two isoforms of DDAH, DDAH-1 and DDAH-2. DDAH-1 is usually found in tissues expressing neuronal NOS while DDAH-2 is predominantly found in tissues containing the endothelial isoform of NOS [36]. Increased plasma levels of glucose, oxidized LDL and homocysteine are associated with decreased levels of DDAH. Furthermore, some conventional cardiovascular risk factors may reduce DDAH activity by increasing oxidative stress [37-40]. Pharmacological inhibition of DDAH increases ADMA concentrations and reduces NO production [41].

Conversely, transgenic DDAH overexpression reduces ADMA levels and increases NO levels [42]. In animal studies DDAH overexpression has been shown to promote endothelial repair after vascular injury [43], to suppress myocardial reperfusion injury [44] and to inhibit ADMA-induced endothelial dysfunction in the cerebral circulation [45]. Interestingly, over-expression of DDAH-1 and DDAH-2 appears to result in very similar phenotypic changes, whereas selective silencing of individual DDAH isoforms results in greatly different biological effects [46]. Thus, silencing of DDAH-1 resulted in increased circulating ADMA levels but no change in endothelium-mediated vasodilation, whereas silencing of DDAH-2 resulted in significantly reduced endothelium-mediated vasodilatation with no concomitant change in plasma ADMA concentration. This finding corresponds to the observation that DDAH-2 is the most abundant isoform in endothelium, whereas DDAH-1 is found at high expression levels in kidneys and liver.

### ADMA and its Role in Endothelial Dysfunction and Cardiovascular Disease

As noted above, ADMA is an endogenous competitive inhibitor of NO synthase, and thus may cause endothelial dysfunction [20, 47]. The link between plasma ADMA levels and established and emerging risk factors for progression of vascular disease has therefore been investigated. Putatively, a number of such risk factors, including obesity [48], hypertension [49], hypercholesterolemia [14], smoking [50], diabetes mellitus [51], hyperhomocysteinemia [52] and vascular inflammation [53] might mediate their deleterious effects on the vascular wall through the dysfunction of the endothelial L-arginine/NO pathway. In each of these conditions, ADMA plasma levels have been shown in case-control studies to be elevated compared to healthy controls.

Local intra-arterial infusion of ADMA can significantly reduce forearm blood flow [47]. Moreover, intravenous infusion of ADMA increased mean blood pressure by 6% and systemic vascular resistance by 24%, while reducing the effect of exercise on cardiac output (by ~15%), heart rate, and vascular responsiveness [54].

### ADMA and Endothelial Function: Clinical Studies

In a randomized, double-blind, placebo-controlled study investigating endothelial function as measured by brachial artery flow-mediated dilatation (FMD), serum ADMA and serum L-arginine levels in 49 hypercholesterolemic individuals were compared to individuals with normal cholesterol levels. People with hypercholesterolemia had impaired endothelial function, increased ADMA levels and decreased L-arginine/ADMA ratio. ADMA levels were inversely correlated to the endothelial-dependent vasodilatation. Furthermore, intravenous infusion of L-arginine normalised not just the L-arginine/ADMA ratio but also normalised the endothelial function [14].

Perticone and colleagues studied serum ADMA levels and endothelial function in people with essential hypertension [55]. People with hypertension had impaired brachial artery FMD and increased ADMA levels. These measures were inversely correlated with ADMA levels independently accounting for 34% of the interindividual variability in peak flow-mediated dilatation. Infusion of L-arginine improved the endothelial function.

In a case control study, Melikian and colleagues studied endothelial function as measured by brachial artery FMD, together with plasma ADMA levels, and ciruculating measures of inflammation and oxidative stress. Black African people were noted to have higher ADMA levels and impaired endothelial function compared to the white Europeans. No difference in C-reactive protein levels was noted between the two groups. In a stepwise multiple regression model, ADMA was the only independent determinant of flow-mediated dilatation (p=0.02) [56].

In the young Finns study involving 2096 white adults, of age 24-39 years, plasma ADMA levels and brachial artery FMD was measured along with other conventional cardiovascular risk factors. There was an inverse correlation between ADMA and FMD. This inverse association between ADMA and FMD remained significant in a multivariate regression model adjusted for age, sex, conventional cardiovascular risk factors, estimated glomerular filtration rate and baseline brachial artery diameter [57].

In a cross-sectional study of 121 non-diabetic people with proteinuria, ADMA levels correlated with the proteinuria as well as with the presence of secondary amyloidosis. Furthermore, ADMA was an independent predictor of FMD [58]. In a study in 24 people with Type 2 diabetes, intensive treatment of diabetes was shown to be associated with reduced serum ADMA levels and this inversely correlated to improved endothelial function [59].

ADMA has been found to modulate coronary endothelial function [60] and promotes coronary spasm in small studies [61]. However, in a randomized, double-blind trial in 289 patients with coronary artery disease, ADMA, L-arginine and coronary endothelial function as assessed by the coronary artery response to local acetylcholine infusion were measured [62]. No correlation between coronary endothelial function and ADMA levels was found. In another study, coronary endothelial dysfunction was shown to be independently associated with erectile dysfunction and plasma ADMA concentration in men with early coronary atherosclerosis [63].

Endothelial function has been implicated as the underlying pathophysiological phenomenon responsible for slow coronary flow (SCF). Serum ADMA and L-arginine levels were measured in 31 people with SCF and compared with age-matched healthy people. People with SCF had significantly higher levels of ADMA and a higher L-arginine/ADMA ratio compared to healthy people. ADMA levels and the L-arginine/ADMA ratio were significantly correlated to mean thrombolysis in myocardial infarction (TIMI) flow levels. On multivariate regression analysis, plasma ADMA levels were found to be independently predictive of SCF [64].

### ADMA and Carotid Intima-Media Thickness (CIMT)

Increased carotid intima-media thickness (CIMT) has been shown to be a surrogate marker for predicting cardiovascular risk. In a study by Miyazaki and colleagues, stepwise regression analysis showed plasma ADMA levels to be significantly correlated to CIMT [15]. In an epidemiological study of 712 people, plasma ADMA levels were measured along with CIMT. On multiple stepwise regression analysis, CIMT was significantly correlated with ADMA levels [65] and they subsequently showed that the progression of CIMT, over a 6 year period, was related to serum ADMA levels [66]. In the PREVENCION study of 922 adult patients, ADMA significantly predicted CIMT even after adjustment for cardiovascular risk factors, C-reactive protein, and renal function, but did not predict carotid-femoral pulse wave velocity, blood pressure, or hemodynamic abnormalities [67]. Kocak and colleagues found higher levels of ADMA in people without known atherosclerotic disease who were on peritoneal dialysis and a significant positive association between the ADMA levels and CIMT in these individuals [68]. 

### ADMA and Cardiovascular Disease: Clinical Studies

A number of studies have shown a relationship between raised ADMA concentrations and cardiovascular disease. Raised plasma ADMA concentrations have been reported in people with coronary artery disease (CAD) [23], peripheral arterial disease [69, 70], chronic heart failure [71], pulmonary hypertension [72], preeclampsia [73], stroke [74], and hypertrophic cardiomyopathy [75].

In a nested case-control study, 88 men with incident coronary events (fatal and nonfatal myocardial infarction and sudden cardiac death) and 254 age-matched controls from the population-based Monitoring of Trends and Determinants in Cardiovascular Disease/Kooperative Gesundheitsforschung in der Region Augsburg study were followed-up for a median of 6.2 years [76]. In this study, increased plasma ADMA levels were found to predict risk for coronary arterial events in non-smokers, but not in smokers. The authors speculated that this difference could be because of an alteration of the ADMA metabolism by a component of tobacco smoke [76].

In a prospective trial of 225 patients undergoing haemodialysis, age and ADMA levels were the strongest predictors of cardiovascular events and mortality after a median follow- up of 33 months [77]. The Coronary Artery Risk Determination investigating the Influence of ADMA Concentration (CARDIAC) study included 800 people with and without established coronary artery disease (CAD). The plasma ADMA concentration was 20% higher in the presence of established CAD (stable angina or MI) and the ADMA levels increased with increasing number of cardiovascular risk factors. The risk of CAD increased by more than two-fold for every 1 µmol/l increase in plasma ADMA [78]. In a prospective, nested, case-control study of middle-aged men from Finland, Valkonen and colleagues reported a 3.9 fold increased risk of acute coronary events in subjects with highest quartile of ADMA compared to other quartiles [23].

Lu and colleagues followed-up 153 people with stable angina undergoing percutaneous coronary intervention for a duration of 16 months during which time major cardiovascular events occurred in 51 patients. An increased risk of cardiovascular events was noted with increasing levels of ADMA which was independent of any confounding factors in a multi-factorial Cox regression analysis [22].

In the Ludwigshafen Risk and Cardiovascular Health (LURIC) study, 2543 people with angiographically documented CAD and 695 with no CAD were followed-up for 5.5 years [24]. Increased all-cause mortality and death due to cardiovascular causes were noted in the second, third and fourth quarters of ADMA when compared to the lowest quarter (hazard ratio of 1.09, 1.40 and 2.04 respectively). However, the predictive value of ADMA was not statistically significant in the subgroup of patients without angiographically proven CAD [24].

In the AtheroGene study, baseline serum concentrations of ADMA were studied in 1874 consecutive patients with CAD who were then followed-up for 2.6±1.2 years. The primary end-point was death from cardiovascular causes or non-fatal myocardial infarction. The median ADMA levels in patients who subsequently experienced the primary end-point was significantly higher than in patients who did not reach the primary end-point. The hazard ratio for the primary end-point was 2.48 times higher in patients whose ADMA was above the highest tertile compared to those in who ADMA was below the lowest tertile [79].

In a prospective study Krempl and colleagues studied ADMA at baseline and 6 weeks post-PCI in patients with CAD (stable angina and unstable angina) compared with healthy individuals. They reported higher ADMA levels in patients with CAD compared to healthy controls. Patients with unstable angina had significantly higher ADMA levels compared to people with stable angina. Patients with unstable angina whose ADMA levels decreased at 6 weeks were found to have less frequent recurrence of cardiovascular events compared to patients whose ADMA levels remained high post-PCI [80].

Baseline ADMA levels were measured in 880 healthy women who were followed-up for 24 years in the Population Study of Women in Gothenburg [81]. The authors reported that a 0.15 μmol/l increase in baseline ADMA levels was associated with an approximately 30% increase in incident cardiovascular risk and 30% increase in fatal cardiovascular disease after adjustment for conventional cardiovascular risk factors, creatinine clearance and homocysteine. Plasma ADMA levels ≥0.71 µmol/l enhanced risk assessment for cardiovascular disease in women beyond that predicted by the SCORE and Framingham criteria [81].

High ADMA levels have been reported to predict death 1 year after an acute myocardial infarction. Using Cox multivariate analysis, ADMA above the highest tertile was a predictor for increased mortality (hazard ratio 4.83), when compared to levels in the lowest third of ADMA, even after adjusting for potential confounders such as acute therapy, biological, and clinical factors [82]. In patients with acute coronary syndrome, elevated baseline levels of ADMA have been shown to be a strong and independent predictor of cardiovascular outcomes, including fatal and non-fatal myocardial infarction, stroke and all-cause mortality [83].

In a case-control study, 32 patients who had received a cardiac transplant underwent intravascular ultrasound of the coronary arteries at one month and one year following the cardiac transplant. Plasma ADMA levels were also measured. Change in intimal volume of the vessel wall greater than the median and vascular remodeling were major outcome measures. Plasma ADMA levels were associated with subsequent development of intimal hyperplasia with a risk ratio of 2.72. ADMA levels did not correlate with negative coronary remodeling. Treatment with sirolimus, as compared with mycophenolate mofetil was associated with significantly lower ADMA levels and less intimal hyperplasia [84]. 

In a prospective study of 496 patients with peripheral arterial disease, ADMA and L-arginine levels were measured at baseline and then after 19 months follow-up. The occurrence of MACE (myocardial infarction, percutaneous coronary intervention, coronary artery bypass graft, stroke, carotid revascularization and death) was evaluated. MACE occurred in 39% patients with the highest quartile of ADMA compared to 26% in patients with lowest quartile of ADMA. There was no association between L-arginine levels and the occurrence of MACE [70].

Post-myocardial infarction cardiogenic shock is associated with increased mortality. Nicholls and colleagues studied ADMA levels in patients with myocardial infarction who were in cardiogenic shock. ADMA levels were not only significantly higher in patients with cardiogenic shock but people with higher levels of ADMA were more likely to die during the 30 days follow-up compared to those who had low levels of ADMA. ADMA remained the only independent predictor of mortality on multiple logistic regression analysis [85].

In a prospective observational study, ADMA levels were investigated as a marker for adverse events in people undergoing non-cardiac surgery. Patients were followed for 30 days after surgery for a predefined composite end point (death, myocardial infarction/acute coronary syndrome, acute heart failure, severe arrhythmia, embolism, or thrombosis). This study reported that elevated plasma ADMA concentrations were independently associated with a higher risk of adverse events in the per-operative and post-operative periods [86].

Type 2 diabetes is a strong risk factor for increased cardiovascular mortality. In a prospective study of 125 people with Type 2 diabetes, ADMA, L-arginine and C-reactive protein (CRP) levels were measured. Occurrence of a cardiovascular event (myocardial infarction, percutaneous coronary intervention, coronary artery bypass grafting, stroke or all-cause mortality) was defined as a composite end-point. ADMA and CRP levels in the highest tertile were associated with significantly increased hazard ratios for incident cardiovascular events of 2.37 and 3.63 respectively [87].

## MODULATION OF ADMA LEVELS BY PHARMACOTHERAPY

### Statin Therapy

Statins are among the most effective medications for reducing cardiovascular risk. Evidence suggests that the beneficial effects of statins may not just be limited to reducing serum LDL-cholesterol but might also be mediated partly by LDL-independent pleiotropic mechanisms such as improvement of endothelial function by increasing the bioavailability of NO. Some studies have shown that high LDL-cholesterol levels were associated with increased ADMA levels [14, 88].

Further studies have been carried out to assess the effect of statin therapy on ADMA levels. In the majority of the studies, no impact on the ADMA levels was observed with statin therapy [89-92]. However rosuvastatin appeared to reduce ADMA levels in one study [93].

Nevertheless, ADMA may have an important role in modulating the therapeutic response to statin therapy, in improving endothelium-mediated vasodilatation. The mechanism of this effect of statins has been shown experimentally to be via up-regulation of NOS gene expression [94]. In a recent study, simvastatin did not enhance endothelial function in people with elevated ADMA levels whereas it did so in those with low ADMA levels. This suggests that although NO synthase may be upregulated by simvastatin in people with high ADMA levels, this may be ineffective in terms of enzyme activity in the presence of high levels of an enzyme inhibitor like ADMA. A combination of simvastatin and oral L-arginine improved endothelial function in people with high ADMA, whereas it did not change the endothelial response to simvastatin in patients with low ADMA [95]. As NO-mediated effects may play a major role in the therapeutic effects of statins, ADMA concentration might then be an important factor that influences the pleiotropic effects of simvastatin.

### Renin-Angiotensin System Blockers and ADMA Levels

Angiotensin converting enzyme (ACE) inhibitors and angiotensin 2 receptor blockers (ARBs) improve endothelial function and NO availability in conditions like CAD, hypertension and diabetes [96-98]. A number of studies have shown ACE inhibitors and ARBs to reduce ADMA levels in people with hypertension, diabetes mellitus and cardiac syndrome X [99-102].

The results of a study in eNOS knockout mice suggests that ADMA may also exert NO-independent effects via upregulation of ACE and augmentation of oxidative stress through angiotensin-1-dependent pathways [103]. A recent study on spontaneously hypertensive rats suggested that reduction in ADMA levels might be involved in the cardioprotective effect of losartan [104].

### Blood Glucose Lowering Medic**a**tions and ADMA Levels

Type 2 diabetes has been associated with increased ADMA levels and cardiovascular risk [87]. ADMA and NO have been found to be significant determinants of insulin insensitivity [105], a common feature of type 2 diabetes.

Thiazolidinediones (rosiglitazone and pioglitazone) improve insulin sensitivity by acting via the peroxisome proliferator-activated receptors [106] and are used as blood glucose lowering agents in Type 2 diabetes. In a study by Wakino and colleagues, pioglitazone decreased plasma ADMA concentration by about 20% in both spontaneously hypertensive rats and in control normotensive Wistar-Kyoto (WKY) rats [107]. This effect was accompanied by the increase in renal expression of DDAH-2 but not DDAH-1 in both strains.

Stuhlinger and colleagues found that rosiglitazone reduced ADMA by 30% in seven insulin-resistant non-diabetic hypertensive individuals [19]. In a more recent study in 20 people with Type 2 diabetes, treatment with rosiglitazone was reported to have improved endothelial function but without reduction of ADMA levels [108].

Metformin is a blood glucose-lowering agent commonly used in type 2 diabetes. In a study by Asgami and colleagues, people with type 2 diabetes were treated with metformin for 3 months. Serum ADMA levels were reduced by 30% while no change in serum SDMA or L-arginine levels was noted [109]. Furthermore, treatment with metformin was found to reduce ADMA levels in young, non-obese, non-hypertensive individuals with polycystic ovarian syndrome [110].

### Aspirin and ADMA

Several studies have suggested a cardio-protective role for aspirin [111]. In a study in rats, low dose aspirin (30 mg/kg) was assessed for its effect on LDL-induced endothelial dysfunction. The results of this study suggested that aspirin at a lower dose (30 mg/kg) protects the endothelium against damages elicited by LDL *in vivo*, and that the protective effect of aspirin on endothelium is related to reduction of ADMA concentration by increasing DDAH activity [112].

ADMA has been reported to accelerate cell senescence [113]. Aspirin has been reported to delay onset of replicative senescence. Along with the delayed onset of senescence, aspirin decreased reactive oxygen species and increased nitric oxide (NO) and cGMP levels. Furthermore, aspirin reduced the elaboration of ADMA and up-regulated the activity of DDAH [114].

### Hormone Replacement Therapy and ADMA

It is well established that the risk of cardiovascular disease increases sharply after menopause in women. A number of retrospective and cross-sectional studies indicate that women treated with estrogen replacement therapy have improved vascular function and a lower incidence of CAD [115].

The effect of subcutaneous oestradiol on ADMA levels was studied in 15 post-menopausal women [116]. The authors reported that estrogens can alter the catabolism and release of ADMA *in vitro* and reduce the circulating concentration *in vivo*. Increased DDAH activity and the subsequent fall in ADMA was proposed to contribute to the positive effect of estrogen on NO synthesis [116].

In a subsequent study on endothelial cells in culture, it was reported that oestradiol completely reverses the effects induced by oxidized LDL on the DDAH/ADMA/NO pathway. This study showed that oestradiol reversed decreased NO release, increased ADMA production and reduced DDAH activity [117].

## CONCLUSIONS

Since its first description as an inhibitor of NO synthesis in 1992, there has been accumulating evidence that ADMA plays an important role as a regulator of NO production in the endothelium. Experimental data from cell culture and animal experiments and cross-sectional studies in humans suggest an association between elevated ADMA concentrations and cardiovascular diseases. Recently *in vivo* models have become available that support the pathophysiological relationship between ADMA and vascular disease, as overexpression of DDAH (which leads to a reduction of circulating ADMA concentration by about 20%) lowers systemic vascular resistance and blood pressure in mice and protects from vascular damage in various disease models, whilst genetic or pharmacological disruption of DDAH (which increases plasma ADMA levels by some 20% in mice) causes hypertension and endothelial dysfunction. The, prospective clinical studies do suggest that ADMA may be a potential diagnostic tool for improved cardiovascular risk assessment, but the integration of ADMA into currently applied risk scores still needs to be performed and validated.

To date, approved pharmacological strategies for cardiovascular risk reduction have led to disappointing results. Most lipid-lowering and blood pressure-lowering drugs have failed to induce significant changes in ADMA concentration. By contrast, oral glucose-lowering medications for diabetes, oestrogens, and ACE inhibitors cause a modest reduction in ADMA. However, as all of these drugs influence cardiovascular risk by other mechanisms, it will be hard to determine whether the change in ADMA induced by these agents does affect cardiovascular disease risk. Specific therapeutic interventions in the DDAH/ADMA pathway are still lacking, and would be needed to perform the necessary randomized controlled trial to assess whether clinical benefit is to be gained by its modulation.
